# Green Synthesis of Nanomaterials and Their Biological Applications

**DOI:** 10.3390/nano11112842

**Published:** 2021-10-25

**Authors:** Giovanni Benelli

**Affiliations:** Department of Agriculture, Food and Environment, University of Pisa, Via del Borghetto 80, 56124 Pisa, Italy; giovanni.benelli@unipi.it; Tel.: +39-050-221-6141

## Introduction

Nanomaterials possess valuable physical and chemical properties, which may make them excellent candidates for the development of new insecticides, acaricides, fungicides, drugs, catalysts, and sensors, to cite just some key categories. To avoid the utilization of toxic or high-energy inputs, which are routinely used in nano-synthesis, the “green synthesis” concept has been proposed, outlining the use of microbial-, animal-, and plant-borne compounds as reducing and stabilizing agents. Even though a large number of studies have been published on the topic, many potential applications of nanomaterials have scarcely been explored, and their real-world applications are poorly implemented. This is often due to a lack of prolonged efficacy of the bioactive compounds and challenging regulations. Nanotechnologies are currently considered a strategic option to improve the efficacy and stability of classic and green insecticides, acaricides, repellents and antiparasitic drugs, relying on various nanocarriers, including nanoencapsulation and nanoemulsions.

In this framework, the journal *Nanomaterials* has already dedicated a successful Special Issue to the topic “*Green Synthesis of Nanomaterials*” in 2019 [1].

The Special Issue “*Green Synthesis of Nanomaterials and Their Biological Applications*” represents a continuation of the former, with another collection of top-quality articles in this research area. Particular attention has been devoted to entomological research, because the widespread overuse of synthetic insecticides leads to the rapid development of resistance in target species, and non-target effects on human health and the environment. A comparable scenario is well recognized in parasitology concerning the use of drugs to manage parasites of public health importance. 

Herein, both original research and reviews have been considered for publication. The present Special Issue includes contributions on the following research topics:

(i) Green-based processes improving the stability of nanomaterials [2];
(ii) The development of green synthesis protocols for the preparation of nanomaterials with antibacterial and anticancer activity, as well as acting as catalytic reductors of nitrophenols [3];
(iii) The development of novel insecticides against key insect pests, including highly stable insecticidal nanoemulsions toxic to moth larvae [4], as well as silica-nanoparticle-coated insecticidal nets effective against aphids and stored-product beetles [5];
(iv) The development of new terahertz metamaterials for biosensing applications [6,7].

The Special Issue ends with a review on cystic echinococcosis [8], a dangerous and hard-to-manage parasitic disease [9,10], summarizing current knowledge on the scolicidal activity of organic and inorganic nanoparticles evaluated through in vitro, in vivo, and ex vivo studies, also considering possible synergistic effects with anti-parasitic drugs currently used.

Overall, I am grateful to all the authors for their fine contributions to the present Special Issue, and hope that the published studies will pave the way for novel real-world applications of green nanomaterials.

## Data Availability

Not applicable.
